# Supplementary material for: Comparison of the Morpho-Physiological and Molecular Responses to Salinity and Alkalinity Stresses in Rice
Source: Plants (Basel). 2023 Dec 23;13(1):60. doi: 10.3390/plants13010060 (PMC10780433; doi:10.3390/plants13010060)
Supplement: Supplementary file 1 [file plants-13-00060-s001.zip › plants-2754369-supplementary.pdf]

Supplementary Table S1. Analysis of variance for ten morpho-physiological parameters in rice genotypes at the seedling stage under both alkaline and saline stress

Trait name	SIS	CHL (SPAD units)	SL (cm)	RL (cm)	SNC (mmol kg^{-1})	SKC (mmol kg−1)	RNC (mmol kg−1)	RKC (mmol kg−1)	SNaK (ratio)	RNaK (ratio)
Genotype (F values)	17.46^{***}	12.08^{***}	35.26^{***}	12.20^{***}	0.94^{ns}	1.60^{ns}	9.43^{***}	5.70^{***}	1.31^{ns}	8.55^{***}
Treatment (F values)	3.85^{ns}	0.001^{ns}	11.87^{**}	9.97^{**}	0.61^{ns}	31.74^{***}	3.20^{ns}	35.08^{***}	37.96^{***}	4.74^{**}
Genotype x Treatment (F values)	4.87^{*}	2.26^{ns}	2.06^{ns}	0.33^{ns}	1.18^{ns}	2.50^{ns}	7.86^{***}	9.07^{***}	0.50^{ns}	4.13^{**}

SIS, salt injury score; CHL, chlorophyll content, SL, shoot length; RL, root length; Trait means are averages of three replications, SNC, shoot sodium concentration; SKC, shoot potassium concentration, RNC, root sodium concentration; RKC, shoot potassium concentration; SNaK, ratio of sodium and potassium concentration in shoot; RNaK, ratio of sodium and potassium concentration in root

*, **, *** indicate significance at 0.05, 0.01, and 0.001 probability level

a, b, c, d represent Tukey lettering for determining difference among genotypes and shared letter between the genotypes indicates no significant difference at 0.05 probability level.

Supplementary Table S2. Trait means for ten morpho-physiological parameters in rice genotypes at the seedling stage under control condition.

Genotype	Pokkali	Geumgangbyeo	Mermentau	Bengal
SIS	1± 0.0	1 ± 0.0	1 ± 0.0	1 ± 0.0
CHL (SPAD units)	32.56 ± 0.64	29.05 ± 1.05	31.96 ± 2.7	32.86 ± 2.2
SL (cm)	47.22 ± 2.9	36.66 ± 2.3	40.38 ± 3.9	45 ± 2.58
RL (cm)	13.72 ± 2.42	10.55 ± 1.17	9.64 ± 1.03	9.66 ± 0.76
SNC (mmol kg−1)	90.47 ± 7.7	45.58 ± 5.46	18.65 ± 2.32	72.52 ± 5.44
SKC (mmol kg−1)	1463.003 ± 89.9	1334.05 ± 101.34	1237.35 ± 123.24	1538.22 ± 121.94
RNC (mmol kg−1)	213.09 ± 10.4	135.54 ± 17.66	95.48 ± 18.82	227.95 ±23.22
RKC (mmol kg−1)	2466.44 ± 168.49	2124.25 ± 102,78	2675.76 ± 112.76	2090.95 ± 132.70
SNaK (ratio)	0.0616 ± 0.003	0.03 ± 0.02	0.01 ± 0.01	0.04 ± 0.009
RNaK (ratio)	0.08 ± 0.01	0.06 ± 0.02	0.03 ± 0.01	0.11 ± 0.02

SIS, salt injury score; CHL, chlorophyll content, SL, shoot length; RL, root length; Trait means are averages of three replications, SNC, shoot sodium concentration; SKC, shoot potassium concentration, RNC, root sodium concentration; RKC, shoot potassium concentration; SNaK, ratio of sodium and potassium concentration in shoot; RNaK, ratio of sodium and potassium concentration in root
